# Alcohol consumption and binge drinking in adolescents: comparison of different migration backgrounds and rural vs. urban residence - a representative study

**DOI:** 10.1186/1471-2458-11-84

**Published:** 2011-02-07

**Authors:** Carolin Donath, Elmar Gräßel, Dirk Baier, Christian Pfeiffer, Deniz Karagülle, Stefan Bleich, Thomas Hillemacher

**Affiliations:** 1 Psychiatric University Clinic Erlangen, Department Medical Psychology and Medical Sociology, Schwabachanlage 6, 91054 Erlangen, Germany; 2 Criminological Research Institute of Lower Saxony, Lützerodestr. 9, 30161 Hannover, Germany; 3 Center for Addiction Research, Clinic for Psychiatry, Social Psychiatry and Psychotherapy, Hannover Medical School, Carl-Neuberg-Str. 1, 30625 Hannover, Germany

## Abstract

**Background:**

Binge drinking is a constant problem behavior in adolescents across Europe. Epidemiological investigations have been reported. However, epidemiological data on alcohol consumption of adolescents with different migration backgrounds are rare. Furthermore representative data on rural-urban comparison concerning alcohol consumption and binge drinking are lacking. The aims of the study are the investigation of alcohol consumption patterns with respect to a) urban-rural differences and b) differences according to migration background.

**Methods:**

In the years 2007/2008, a representative written survey of N = 44,610 students in the 9^th. ^grade of different school types in Germany was carried out (net sample). The return rate of questionnaires was 88% regarding all students whose teachers respectively school directors had agreed to participate in the study. Weighting factors were specified and used to make up for regional and school-type specific differences in return rates. 27.4% of the adolescents surveyed have a migration background, whereby the Turkish culture is the largest group followed by adolescents who emigrated from former Soviet Union states. The sample includes seven large cities (over 500,000 inhabitants) (12.2%), independent smaller cities ("urban districts") (19.0%) and rural areas ("rural districts") (68.8%).

**Results:**

Life-time prevalence for alcohol consumption differs significantly between rural (93.7%) and urban areas (86.6% large cities; 89.1% smaller cities) with a higher prevalence in rural areas. The same accounts for 12-month prevalence for alcohol consumption. 57.3% of the rural, re-spectively 45.9% of the urban adolescents engaged in binge drinking in the 4 weeks prior to the survey. Students with migration background of the former Soviet Union showed mainly drinking behavior similar to that of German adolescents. Adolescents with Turkish roots had engaged in binge drinking in the last four weeks less frequently than adolescents of German descent (23.6% vs. 57.4%). However, in those adolescents who consumed alcohol in the last 4 weeks, binge drinking is very prominent across the cultural backgrounds.

**Conclusions:**

Binge drinking is a common problem behavior in German adolescents. Obviously adolescents with rural residence have fewer alternatives for engaging in interesting leisure activities than adolescents living in cities. This might be one reason for the more problematic consumption patterns there. Common expectations concerning drinking behavior of adolescents of certain cultural backgrounds ('migrants with Russian background drink more'/'migrants from Arabic respectively Oriental-Islamic countries drink less') are only partly affirmed. Possibly, the degree of acculturation to the permissive German alcohol culture plays a role here.

## Background

Problematic alcohol consumption patterns including binge drinking is a constant evi-dent behavior in many adolescents across Europe and the USA [1-3]. Aside from the direct con-sequences of intoxication and its possible somatic complications, the long-term consequences of this consumption pattern are disadvantages in different social areas of life (school, education, job perspectives; risky behavior in traffic and sexual activity [4,5]; delinquency [6]) and according to the latest research also biological changes in neuronal processes of the hippocampus likely resulting in memory and cognitive deficits [7]. Often the drinking behavior is associated with certain cultural or seasonal events, like "Spring Break" in the U.S.A., certain folk festivals like the Oktoberfest in Germany, "Botellóns" in Spain, or internet-organized (using social networks) spontaneous drinking parties on public places in France. However, it seems, according to epidemiological data, that excessive alcohol consumption is not limited to one or two events per year but is a regular leisure time activity for many adolescents and university students [8]. The 2007 ESPAD report (European survey of 15-/16 year-olds concerning substance use in 35 European countries) states that heavy episodic drinking (having had 5 or more drinks on one occasion in the last 30 days) varies across Europe between 20% (Iceland) and 60% (Denmark). No data are reported for Germany. Except for the north-western part of Europe, boys more often consume heavily on any one occasion than girls [9,10]. The German Federal Center for Health Education regularly carries out a representative survey of 12- to 17-year olds concerning their substance consumption. The 2008 data concerning alcohol consumption show that alcohol was the most-widely used psychoactive substance: three-fourths of the adolescents stated its use. 17.4% of the adolescents consume alcohol weekly or more often, again boys in a greater proportion than girls [11]. Binge drinking (same definition as in the ESPAD-study) is reported by 20.4% of the 12-to-17-year-olds. The "Child-and-Youth-Health-Survey" carried out by the Robert Koch Institute on behalf of the German Ministry of Health [12] also reflects for the first time in a representative sample selective aspects of the consumption patterns of adolescents with migration background. Concerning alcohol, the consumption frequency is reported as regular use. While 40.8% of the 11-17-year-olds without migration background regularly consume alcohol (at least once a week), adolescents with one-sided or two-sided migration background do this more rarely (34.0% respectively. 17.9%). Because of the lack of more detailed data concerning alcohol consumption patterns in adolescents with migration background in Germany in the representative studies cited, a closer look at it will be taken in this work. Therefore, the first question which will be investigated in this work is: How can consumption patterns including binge drinking be described in adolescents with migration background in comparison to German adolescents? Furthermore, none of the above-mentioned representative data collections reports on possible consumption differences between rural and urban residence of the adolescents. However, it is known that environmental influences can play a role in the development of problem drinking behavior [13]. Therefore, as a second question, consumption patterns are investigated in this work from the perspective of possible rural-urban differences.

## Aims

I) Description of consumption patterns with respect to possible differences between urban and rural residence

II) Description of consumption patterns in adolescents with different migration backgrounds living in Germany

## Methods

### Design

The matter concerns a representative survey of 9^th^-graders in Germany which was carried out in 2007/2008. In the year 2006, there were 910,000 9^th^-graders in Germany. The goal was to survey 50,000 adolescents from different regions. The basis for the selection of the regions was the federal classification of rural districts and independent cities (urban districts) which total up to 440. The latter contain cities of each size (below 100,000 up to 3.3 million (Berlin) inhabitants). The number of inhabitants in the rural districts also varies from about 50,000 to over 600,000. Therefore, the rural and urban districts (= regions) were sorted into classes of region size in which the random drawing then took place. The classes of region size were: Western Germany (urban districts): cities with more than 500,000 inhabitants, cities with more than 100,000 inhabitants, cities with less than 100,000 inhabitants; Western Germany (rural districts): districts with more than 100,000 inhabitants, districts with less than 100,000 inhabitants; Eastern Germany (former GDR) (urban districts): cities with more than 100,000 inhabitants (there are only two cities with more than 500,000 inhabitants), cities with less than 100,000 inhabitants; Eastern Germany (former GDR) (rural districts): districts with more than 100,000 inhabitants, districts with less than 100,000 inhabitants; special case: Berlin. With the knowledge about the number of 9^th^-graders in each class of region size (from the official education statistics) and the goal of 50,000 adolescents to be questioned, it was possible to calculate how many adolescents per class of region size had to be included. It has to be taken in account that not students but classes were drawn by chance. The number of 50,000 students refers to a goal of 2,500 classes. It is known that about 20 students per class can be retrieved and used for data analysis. The number of 2,500 classes was chosen in the way that for every region in Germany which was supposed to be represented in the survey a sufficient number of classes was evident. The goal was to display the distribution of the 9^th^-graders in the classes of region size (in the population) to the same percentage in the sample. It was assumed that every 2^nd ^student (in large cities every 6^th ^student) in a drawn region would be questioned. Thus it could be calculated how many re-gions had to be drawn out of every class of region size. These steps resulted in 61 regions. Which region was chosen to take part was then drawn by chance in order to secure a representative sample. At the Criminological Research Institute of Lower Saxony, the sample was drawn stratified to school type (on basis of school lists provided by the local education authorities). A master list on which all school classes (9^th^grade) of one region were consecutively sorted was used. Then all directors of the drawn schools were informed in writing about the survey and asked for participation of their 9^th^-grade school classes. If the directors agreed to the survey, information material including consent forms for parents were sent to the schools. On a concerted appointed day, the written survey was carried out without the students whose parents refused participation, who themselves refused to participate or who were otherwise busy respectively absent during the survey. The survey at the school was carried out by trained external study assistants - not by the employees of the schools - in order to preserve reliability and validity. A detailed description of the design and implementation of the study is published elsewhere [14].

The research project was granted by the Federal Ministry of the Interior in Germany, a statement of an ethics committee was not necessary. Instead the survey was audited by each Ministry of Education of every German state (Bundesland) and additionally of every state responsible for data protection. Only in those states where through this procedure the survey was permitted the survey then actually took place. A further ethics committee was not included since the data protection matters were covered by the above described procedure and another intervention besides filling out an anonymous questionnaire was not applied.

### Instruments

Substance consumption was investigated substance- (and beverage-) specific with items used in a representative survey of the Criminological Research Institute of Lower Saxony in 2001 [15]. In this work, only data concerning alcohol, including the beverages beer, wine/sparkling wine, alcopops and hard liquor, are presented. The adolescents were asked if they had a) tried the specific substance already once in their lifetime, b) how old they were when they did that and c) how often they had consumed the specific substance during the last twelve months. The answer categories were: a) yes/no, b) 6, 7, 8, (...), 20 years or older and c) never, 1 to 12 times a year, several times a month, once or more per week, daily. The item assessing heavy episodic drinking (binge drinking) was derived from the representative survey of adolescents of the German Federal Center for Health Education [16]. Binge drinking is defined as the consumption of five or more standard drinks at one drinking opportunity. The adolescents were asked a) if they had consumed alcohol in the last 30 days (30-day-prevalence) and if yes, b) on how many days they had consumed 5 or more standard alcoholic drinks in a row. The answer categories were a) yes/no and b) not on one day, on one day, on two days, (...), on 20 or more days.

The urban-rural comparison is based on the following definition: large cities (> 500,000 inhabitants), independent urban districts (smaller cities with more than 100,000 or less than 100,000 inhabitants) and rural districts. Data concerning residence were obtained through the sampling method and did not need to be included in the questionnaire. Migration background was defined as having at least one parent who was born outside of Germany or having been born outside of Germany oneself or having non-German citizenship or having at least one parent of non-German citizenship. The birth place and citizenship of the adolescent and its parents were included in the questionnaire. In the case of discrepancies between the citizenship of mother and father, the status of the mother was used.

### Sample

**Figure 1:**
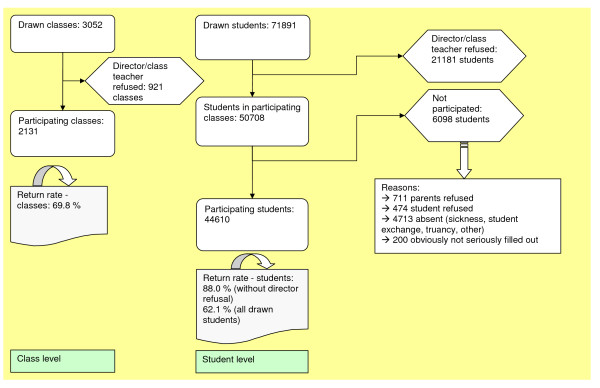
**Sample constitution**.

A total of 3052 classes (9^th ^grade) with 71,891 students were drawn. For 921 classes (21,181 students) the directors/main class teachers refused to participate. 2,131 classes participated with a total of 44,610 students. Actually the 2,131 classes included 50,708 students, but 6,098 of them did not participate (reasons for example: parents refusal or absenteeism). Figure 1 comprises a detailed flow-chart on the sample record.

The return rates (students, without director refusal) differed between the school types in that grammar/secondary schools as well as private/not state-run schools had the highest return rates (92.0/92.8) and special schools the lowest (75.5). Furthermore, the return rates differed between the classes of region size. In the large cities, the return rate was lower in comparison with rural districts and urban districts with less than 500,000 inhabitants. In spite of the varying return rates in the different classes of region size, the realized sample represents the proportions of the population very well (for example students living in cities with more than 100,000 inhabitants in Western Germany: 12.04% in the sample and 11.68% in the population). The proportion of students in the 9^th ^grade in every class of region size in West and Eastern Germany was compared to their proportion in the sample. With those two percentages for each category the reliability can be seen and rated. The proportions never differed more than .36% between population and sample in the different classes of region size except for Berlin where the difference was .62%.

In consequence of the varying return rates, weighting factors were calculated so that the proportion of school forms in the sample corresponds to that in the population and in the same manner, the proportion of regions with different sizes in the sample corresponds to that in the population. The two weighting factors were multiplicatively connected when data of the total sample were analyzed. Thereby the imbalances regarding the school forms were eliminated, as were the much smaller imbalances regarding the classes of region size.

The sample can be characterized as follows: 51.3% of the sample are male students, the mean age is 15.3 (SD 0.7) years. The percentage of adolescents with migration background is 27.4, whereby students with a Turkish migration background constitute the largest group (6.0%; more than 2,600 students) followed by emigrants from the former Soviet Union states (5.8%; more than 2,500 students). A total of 12.2% lives in large cities with more than 500,000 inhabitants including Berlin while the majority lives in rural districts (68.8%). The migration background varies between 39.9% in large cities with more than 500,000 inhabitants and 23.9% in rural districts.

### Statistical analysis

Prevalence analyses were carried out with descriptive statistics. Group differences were analyzed according to the measurement level of the variable with ANOVAs (continuous variables) respectively Chi^2^-tests (categorical variables). SPSS 17.0 was used. Questions concerning substance consumption beyond life-time use were only analyzed for adolescents with positive life-time prevalence (who answered "yes" to the question if they had ever tried alcohol). Other staged questions were handled in the same manner. Because of the sample size, the level of significance was set to p = .001 [17]; exceptions are mentioned. A sensitivity analysis was carried out to disentangle the rural-urban differences from the different proportions of adolescents with migration background living there. Rural-urban differences concerning life-time-prevalence and 12-month-prevalence for alcohol in general and the different alcoholic beverages were additionally explored with German adolescents only (without migration background). Again, Chi^2^-tests were used to explore statistically significant differences in the prevalence. The aim of the sensitivity analysis was to confirm statistical differences between urban and rural consumption patterns detected in the whole sample by only looking at the German adolescents so that the difference cannot be attributed to adolescents with migration background.

## Results

### Alcohol consumption patterns in a rural-urban comparison

**Figure 2:**
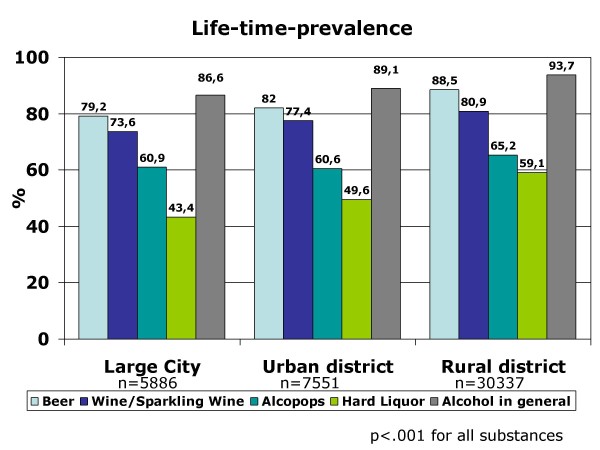
**Life-time prevalence of consumption of alcoholic beverages and alcohol in general: urban-rural comparison**.

**Figure 3:**
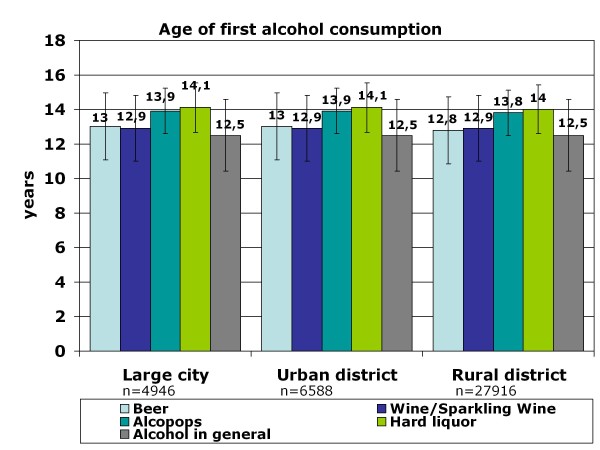
**Age of first alcohol consumption: urban-rural comparison**.

Life-time prevalence for alcohol differs significantly (p < .001) between rural districts (93.7%) and urban areas (86.6% for large cities/89.1% smaller cities). It varies according to the beverage: the highest life-time prevalence exists for beer (86.1%), the lowest for hard liquor (55.2%) independent of place of residence. However for every alcoholic beverage which was investigated, the life-time prevalence is higher in rural areas (p < .001) (see Figure 2). The age of first alcohol consumption differs descriptively by about 0.2 respectively 0.1 month between urban and rural residence for the beverages beer (F (2) = 14.91; p < .001) and alcopops (F (2) = 14.03; p < .001). Adolescents living in rural areas consume these beverages earlier for the first time in their life than adolescents who live in smaller or large cities. There is no significant difference in the age of first consumption of alcohol in general (p = .137), for wine/sparkling wine (p = .206) and for hard liquor (p = .076) which can be seen in Figure 3 that includes the mean values and standard deviations of age of first consumption (Figure 3). The 12-month prevalence for alcohol in general and for each beverage differ significantly (p < .001) between urban and rural adolescents. For example, the percentage of never-consumers is highest in large cities and lowest in rural districts (see Table 1). Furthermore, the percentage of 9^th^-grade stu-dents who regularly consume alcohol (once a week or more often) is considerably higher in rural areas: about one-fourth (24.4%) versus 19.1% in large cities and 20.0% in smaller cities (= urban districts). This is not only true for alcohol in general but can be observed for every beverage.

**Table 1:** 12-month prevalence of consumption of alcoholic beverages and alcohol in general: urban-rural comparison

Beverage	12-month-prevalence	N	Large city (%)	Urban district (%)	Rural district (%)	p (Chi^2^)
Beer	Never	8668	27.7	25.2	17.3	< .001
		
	1-12 times/year	16458	37.5	38.8	38.0	
		
	Several times/month	9135	18.1	18.4	22.4	
		
	1 or several times/week	8527	15.7	16.8	21.2	
		
	daily	456	1.0	0.8	1.1	

Wine/ Sparkling Wine	Never	12058	33.7	30.4	26.2	< .001
		
	1-12 times/year	20834	54.8	56.6	59.9	
		
	Several times/month	4102	7.9	8.7	10.0	
		
	1 or several times/week	1600	3.5	4.1	3.6	
		
	daily	82	0.2	0.2	0.2	

Alcopops	Never	17406	43.0	43.7	39.0	< .001
		
	1-12 times/year	15699	35.6	35.6	36.8	
		
	Several times/month	6442	13.2	12.7	15.8	
		
	1 or several times/week	3441	7.8	7.5	8.0	
		
	daily	158	0.5	0.4	0.3	

Hard liquor	Never	20999	60.8	54.3	44.9	< .001
		
	1-12 times/year	14868	27.9	32.1	36.3	
		
	Several times/month	4653	7.0	8.5	12.1	
		
	1 or several times/week	2537	4.1	4.8	6.5	
		
	daily	103	0.2	0.2	0.3	

Alcohol in general	Never	5557	18.6	16.5	10.7	< .001
		
	1-12 times/year	17755	42.0	43.0	39.8	
		
	Several times/month	10301	20.3	20.6	25.0	
		
	1 or several times/week	9488	17.8	19.0	23.1	
		
	daily	540	1.3	1.0	1.3	

A sensitivity analysis was carried out to disentangle the rural-urban differences from the different proportions of adolescents with migration background living there. Rural-urban dif-ferences concerning life-time-prevalence and 12-month-prevalence for alcohol in general and the different alcoholic beverages were additionally explored with German adolescents only (without migration background). The urban-rural differences were obvious in the same direction and mostly statistically significant: The life-time prevalence for all alcoholic beverages is higher in adolescents with rural residence in comparison to urban residence: beer 90.8% vs. 86.7% (p < .001); wine/sparkling wine 82.0% vs. 84.4% (p = .002); alcopops 64.6% vs. 67.1% (p = .003); hard liquor 50.6% vs. 63.6% (p < .001). The 12-months prevalence is also higher for every alcohol beverage in adolescents with rural living background (p ≤ .001).

The proportion of adolescents who engaged in binge drinking (≥5 drinks on one occasion) in the preceding 30 days is 45.2% for large cities, 46.7% for smaller cities and 57.3% for rural areas, which results in a statistically significant difference (p < .001). Regarding only those adolescents who stated to have consumed alcohol in the last 30 days, the percentage of binge drinking is higher: 74.5% for large cities, 72.9% for smaller cities and 78.4% for rural areas. That means if adolescents do drink alcohol, they tend to drink many drinks on one occasion rather than consuming in a moderate manner. Those adolescents that engage in binge drinking are doing that on 4 to 5 of 30 days on average. Adolescents living in rural areas show that behavior slightly more often (4.68 days vs. 4.45 days in large cities) (F (2) = 25.04; p < .001).

### Alcohol consumption patterns in adolescents with migration background

**Figure 4:**
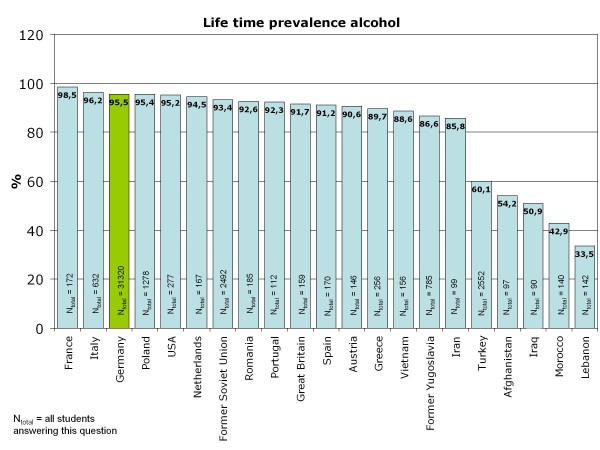
**Life-time prevalence alcohol consumption for all cultural groups of the sample**.

**Figure 5:**
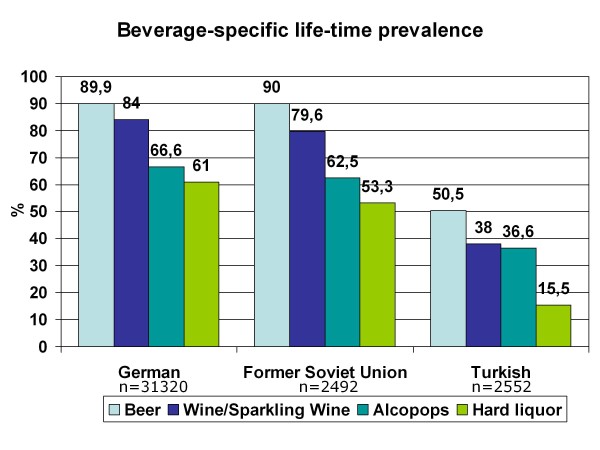
**Beverage-specific life-time prevalence for adolescents with German, Turkish and former Soviet Union cultural background**.

**Figure 6:**
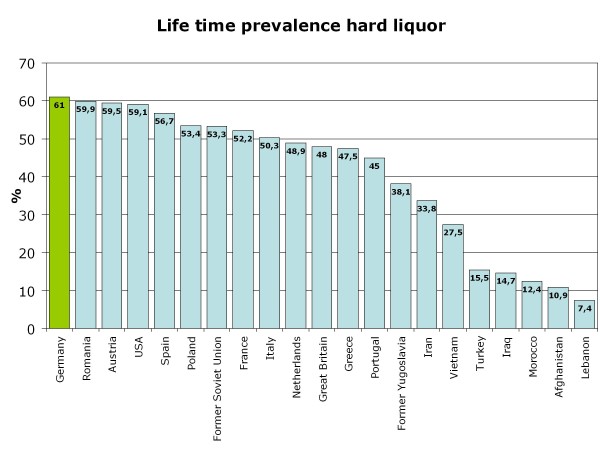
**Life-time prevalence for consumption of hard liquor in 15-year-olds - comparison according to migration background**.

**Figure 7:**
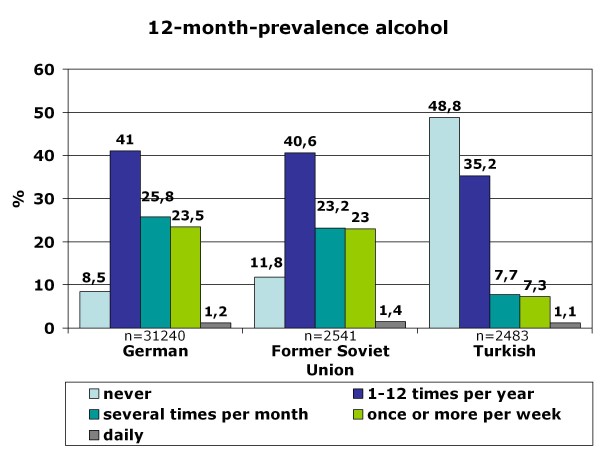
**12-month-prevalence of alcohol consumption for adolescents with German, Turkish and former Soviet Union cultural background**.

**Figure 8:**
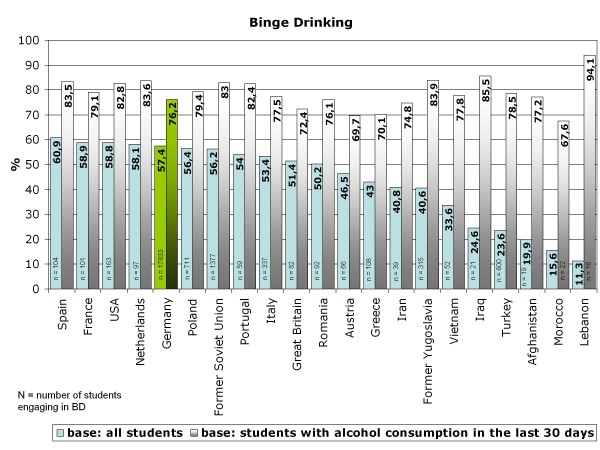
**Binge drinking in 15-year-olds - comparison according to migration back-ground**.

Life-time prevalence for alcohol varies according to the cultural background of the adolescents who migrated to Germany and differs in part substantially from that of German 15-year-olds (Figure 4). As expected, adolescents from Islamic countries have lower life-time prevalence than German or Western European adolescents, except for students from Iran. The three largest groups of 15-year-old students in Germany are compared for the life-time prevalence of specific alcoholic beverages: Germans, adolescents with Turkish migration background and students who emigrated from the former Soviet Union states. As shown in Figure 5 beverage specific life-time prevalence shows the same pattern as life-time prevalence of alcohol with differences between Turkish adolescents and more similar behavior among German and former Soviet Union adolescents. In all three cultural groups, beer is the most commonly tried beverage followed by wine/sparkling wine and alcopops. Hard liquor is the least often tried alcoholic beverage. However, German adolescents have the highest beverage-specific life-time prevalence of the three groups and for hard liquor even of all nations included in the study (Figure 6). The age of first consumption of alcohol in general among German adolescents is 12.50 years (SD 1.98), in adolescents with Turkish background 13.34 (SD 2.01) and adolescents with Russian associated background 12.21 (SD 2.54). This difference is statistically significant (F (2) = 147.85; p < .001). Beverage specific analyses show that basically the youth tries first beer, later wine and sparkling wine, then alcopops and last hard liquor. For German adolescents, there is the exception that the age at first consumption of wine and of beer is nearly the same (12.91 years (SD 1.83) and 12.93 years (SD 1.85)). German adolescents try after beer and wine alcopops at an average age of 13.85 (SD 1.26) years and hard liquor at an average age of 14.02 years. Students with "Russian" migration background try beer with 12.52 (SD 2.47) years, wine respectively sparkling wine with 12.96 (SD 2.17) years, alcopops at an average age of 13.95 (SD 1.57) and hard liquor as the latest beverage at an average age of 14.12 (SD 1.71) years. In contrast, Turkish adolescents try beer at an age of 13.45 (SD 2.04), later wine or sparkling wine at an average age of 13.66 (SD 1.84), alcopops with 14.15 (SD 1.49) years and hard liquor at an average age of 14.08 (SD 1.74). Except for hard liquor the beverage specific first consumption age differs significantly between migration backgrounds as ANOVAs have shown (p < .001 for beer, wine, alcopops). However, the first consumption age of hard liquor is not significantly different between adolescents from different cultural backgrounds (F (2) = 3.54; p = .029). Comparison of the three cultural groups concerning the 12-month prevalence of alcohol consumption shows that almost one-fourth (24.7%) of the German 15-year-olds, less than 10% of the adolescents with Turkish roots (8.4%) and about one-fourth (24.4%) of the adolescents from the former Soviet Union drink alcohol regularly - at least once a week (Figure 7). Beverage-specific 12-month prevalence for the three most common cultural groups in the sample are shown in Table 2. There are significant differences between the three groups for each beverage (p < .001); adolescents with "Russian background" have the highest prevalence of regular consumption (at least once a week) of hard liquor (7.7%), alcopops (9.1%) and wine/sparkling wine (4.9%) followed closely by the German adolescents with very analogical drinking behavior who have the highest prevalence for regular beer consumption (at least once a week) of the three groups (22.5%). The proportion of adolescents who engaged in binge drinking (≥5 drinks on one occasion) in the last 30 days is 57.4% for German 15-year-olds, 23.6% for adolescents with Turkish roots and 56.2% for former Soviet Union emigrants. Looking at the broad range of nations included in the sample, it is evident that binge drinking is most common in adolescents with Western European or North-American background. As to be expected in adolescents who have cultural roots in Islamic-imprinted countries, binge drinking is less evident in general. A different picture opens, though, if one looks only at the adolescents who stated to have consumed alcohol in the last 30 days. Here the adolescents with the two highest binge drinking prevalence are descendents of Oriental-Islamic parents, meaning that if adolescents from these countries do consume alcohol, they almost always do it excessively. But also the descendents of Western, Eastern and South-European parents have a high prevalence of binge drinking when only consumers in the past 30 days are considered (for example former Yugoslavia, Netherlands, Spain) (see Figure 8). The frequency on how many days binge drinkers engage in this risky behavior varies significantly between binge drinkers from different cultural backgrounds (F (21) = 2,526; p < .001). The range is between 2.83 and 6.57 days in the past 30 days. Looking at the three biggest groups in the sample, it is obvious that German students and adolescents from the former Soviet Union act similarly, engaging in binge drinking on 4.58 (SD 4.13) or respectively 4.54 (SD 4.35) days in the last month. The 15-year-olds with Turkish roots who admit to engage in binge drinking had done so on 5.09 (SD 5.51) days during the last month. The three groups do not differ statistically significantly in this variable.

**Table 2:** Beverage-specific 12-month-prevalence for adolescents with German, Turkish and former Soviet Union cultural background

Beverage	12-month-prevalence	N	German roots (%)	Turkish roots (%)	Former Soviet Union roots (%)	p (Chi^2^)
Beer	Never	6741	15.6	58.0	18.5	< .001
		
	1-12 times/year	13269	38.6	29.3	39.9	
		
	Several times/month	7871	23.3	6.2	20.5	
		
	1 or several times/week	7316	21.5	5.8	19.8	
		
	daily	365	1.0	0.6	1.2	

Wine/Sparkling Wine	Never	9558	23.0	69.6	28.8	< .001
		
	1-12 times/year	21422	62.5	26.7	57.5	
		
	Several times/month	3519	10.5	2.2	9.1	
		
	1 or several times/week	1340	3.8	1.3	4.7	
		
	daily	64	0.2	0.1	0.2	

Alcopops	Never	14288	37.3	67.7	42.9	< .001
		
	1-12 times/year	13208	38.2	22.3	34.0	
		
	Several times/month	5439	16.0	5.3	14.0	
		
	1 or several times/week	2830	8.2	4.1	8.7	
		
	daily	124	0.3	0.5	0.4	

Hard liquor	Never	16746	43.0	86.9	51.8	< .001
		
	1-12 times/year	12845	38.4	9.4	29.9	
		
	Several times/month	4078	12.2	1.9	10.6	
		
	1 or several times/week	2144	6.2	1.8	7.5	
		
	daily	77	0.2	0.2	0.2	

Alcohol in general	Never	4192	8.5	48.8	11.8	< .001
		
	1-12 times/year	14726	41.0	35.2	40.6	
		
	Several times/month	8836	25.8	7.7	23.2	
		
	1 or several times/week	8083	23.5	7.3	23.0	
		
	daily	435	1.2	1.1	1.4	

## Discussion

This study uses representative data to generate insight into the variety of substance consumption patterns of 15-year-old adolescents living in Germany. The main focus of the analyses was the comparison of adolescents living in rural and urban settings, and furthermore the comparison of adolescents with different migration backgrounds in contrast to "native" German 9^th^-graders.

Obviously there are differences in alcohol consumption between the areas of residence. Adolescents from rural areas were more rarely "never-drinkers" with respect to having tried alcohol at least once in their life-time and also relating to their drinking behavior in the last 12 months. Even though this might be partly explainable by the higher proportion of adolescents with migration background living in cities, migration background does not seem to be a sufficient factor to explain the difference. Sensitivity analyses showed that the life-time-prevalence and 12-month prevalence also differ significantly between rural and urban areas if only "German" adolescents are taken into account. Furthermore, the differences were also shown in a multivariate analysis of the sample for drugs in general. This constituted a regression analysis with consumption as dependent variable. The control variable urban/rural turned out to be significant [18].

Migration background seems to be reflected in the proportion of never-drinkers during the life-time and during the last 12 months, especially in adolescents with Arabic respectively Oriental-Islamic roots. However, if adolescents with migration background do drink at all, they engage quite often in excessive consumption patterns just like their German counterparts. This is also true for the adolescents with roots in Islamic-imprinted countries who admit to have consumed alcohol lately. In summary, 15-year-olds residing in rural areas drink on more occasions a year, engage more often in binge drinking and have higher life-time prevalence for all alcoholic beverages than 15-year-olds residing in urban areas. The age of first consumption of alcohol does not vary significantly though. One hypothesis for the urban-rural difference could be that in rural areas less multifaceted leisure time activities are available, so that a certain part of the adolescents engages more often in regular alcohol consumption. A study with hints of different consumption types ("TV-type", "party-type", "culture-type", "sport-type") would support this, describing that the "TV-type" drinks more often because of boredom and because of problems [19]. Furthermore in rural areas, cultural traditions are still more strongly anchored and cultural events are celebrated by a broad community of inhabitants. In Germany such cultural events (like country fairs) are traditionally strongly connected with alcohol consumption. Alcohol consumption is positively connotated, Germany has a permissive-drinking culture. Since environmental and societal factors play an important role in influencing the drinking behavior of adolescents [20] and young adults [21], the existing consumption patterns are actually no surprise. Especially since it is known that parental drinking behavior [22] and parental attitudes [23,24] toward alcohol consumption influence the alcohol consumption patterns of the adolescents.

Concerning the consumption patterns of adolescents with migration background, it has to be stated that, as expected, adolescents from countries with Arabic respectively Muslim culture drink to a significantly smaller proportion than German or other Western adolescents. Results in this direction were also reported in the KIGGS-survey [12]. Contrary to expectations, adolescents from the former Soviet Union, which is also known for its permissive drinking culture especially concerning hard liquor, do not differ substantially from German adolescents in their drinking behavior. To the contrary: Germans have the highest life-time prevalence for hard liquor compared to all other cultural backgrounds. However, adolescents from the former Soviet Union have the highest regular consumption (at least once a week) of hard liquor, alcopops and wine compared to German adolescents and adolescents with Turkish roots. But the Germans have the highest prevalence of regular beer consumption. Obviously culture-specific preferences for certain alcoholic beverages stay stabile and do not change in the adopted country. Another hint for that can be seen in the highest life-time prevalence for wine in French and Italian 15-year-olds compared to all other nations which was observed in an additional sensitivity analysis.

Looking at excessive drinking behavior, the prevalence varies enormously when all adolescents from all nations are included. Still, more than every second 15-year-old student from basically all West- and East-European countries respectively from the USA engaged in binge drinking in the last 30-day period. A different extent of binge drinking is only seen in adolescents who emigrated from continents other than Europe or North America. The picture changes when looking only at those who admitted to having consumed alcohol in the last 30-days. Of those adolescents, only a marginally small proportion drinks alcohol in a controlled, not excessive manner. Regardless of cultural background, at least two-thirds of those adolescents engaged in binge-drinking at least once, while more than 3/4 of the German adolescents did so. It is striking that excessive drinking behavior is more dominant than the rather culturally-accepted restricted drinking behavior in adolescents who do drink alcohol. Norman et al. [25] give some explanations for this. Obviously, regular drinking seems to be associated with binge drinking but this alone is not a sufficient explanation. Attitudinal determinants and situational triggers seem to be of importance, too. Situational triggers which lower the inhibition of binge drinking behavior are for example events such as celebrations or being at a party. Binge drinkers report feeling under social pressure ("drinking pressure") from friends in those situations, whereby male binge drinkers seem to have this impression more often than females. Tucker et al. [24] showed that living in a permissive household concerning alcohol and drug consumption has an impact on extensive drinking behavior. Three-fourths of the 9^th^-graders in this study who lived with permissive parents engaged in heavy drinking [24]. But not only permissive attitudes of parents but also positive attitudes towards binge drinking of the adolescents themselves and their expectation of positive consequences is obviously associated with the behavior [25].

The analyses of the study are based on a large representative sample of adolescents which suggests validity of the data. The response rate is lower than in the comparable German part of the ESPAD-study, a representative study with 12,448 participating students at the age of 15/16 years. The reasons are clearly identifiable: In the ESPAD-study school classes were drawn to participate in a second round as substitute for all the classes which were as a whole not willing to participate (teacher/head of the school decision). This has not taken place in the here reported study. Therefore looking only at the response rates of those students, whose teachers/head of schools decided to participate one can perceive that the response rates are relatively similar to the ESPAD study (88.0% our study; 90.4% ESPAD study). However, it has to be taken into account that the adolescents who refused to participate probably engage at least to the same percentage as their participating colleagues in alcohol consumption, maybe even more, since they were unwilling to disclose this behavior. It could therefore be possible that the number of consumers respectively binge drinkers is even underestimated. This should lead to an even stronger focusing on the need for prevention measures and legal regulations which function in the sense of structural prevention. When interpreting the consumption patterns of the different migration groups, one has to consider that some groups are relatively small (less than 100 students) and get even smaller if only a small percentage of those engage for example in binge drinking. Results of these small groups should not be over-interpreted. Furthermore, it has to be taken into account that this was a cross-sectional study. It does not allow predicting substance consumption over time. This would be a very interesting and new aspect to know, especially since data on substance consumption in different migration groups in adulthood are rare.

A definite need for research lies in the possible starting points and kinds of effective prevention measures of problematic alcohol consumption behaviors including primary and secondary prevention. From the research on tobacco prevention it is known that structural prevention measures play an important role in primary and also secondary prevention of smoking behavior [26-28]. To prove the efficacy of those structural measures for adolescents living in a permissive drinking culture in Germany would be an important research task.

In conclusion, the study showed that binge drinking is a prevalent but not the only problematical drinking behavior in 9^th^-graders in Germany. The majority of students was under 16 and actually officially not allowed to buy (and therefore consume) alcohol. However the life-time prevalence of close to 100% at this age (except for Muslim adolescents) shows that alcohol consumption under the legal age is not at all uncommon. Problematical is also the percentage of adolescents who regularly consume alcohol. About one-fourth are likely to develop, respectively continue, risky alcohol consumption in their adult life with possible alcohol-related problems (social, medical, etc.) [29]. The health consequences that are risked range from brain damage [30], damage to the gastrointestinal tract and cardiovascular system up to long-term effects on blood-pressure, in development of cancer and muscular-skeletal disorders [31]. Thus, prevention measures should cover several risky consumption patterns - binge drinking and regular consumption - in order to prevent alcohol related disorders in adult life.

## Conclusions

A representative analysis showed that problematic alcohol consumption patterns (binge drinking, regular weekly or daily consumption) are more common in adolescents living in rural than in urban settings. Prevention measures should therefore not only incorporate individual approaches (behavior-oriented prevention) but also environmental aspects (structural prevention). In the best case, parents should also be targeted concerning their attitudes towards their children's alcohol consumption. This leads to complex prevention approaches which are the ones recommended by experts but which are also expensive and difficult to implement in practice. The complex prevention measures should include focusing on culture-specific attitudes towards alcohol consumption, towards specific beverages and critically reflect the grade of societal permission of alcohol consumption.

## Competing interests

The authors declare that they have no competing interests.

## Authors' contributions

CD carried out data analysis and drafted the manuscript. DB worked out the "Methods" section. CP planned and organized the data collection and revised the manuscript. DK contributed to the background of the manuscript and carried out a literature search. EG contributed to the discussion and the structure of the results section. TH and SB organized the study and helped with the data transfer. TH revised the manuscript. All authors read and approved the final manuscript.

## Pre-publication history

The pre-publication history for this paper can be accessed here:

http://www.biomedcentral.com/1471-2458/11/84/prepub
